# Blue Light Alters the Composition of the Jejunal Microbiota and Promotes the Development of the Small Intestine by Reducing Oxidative Stress

**DOI:** 10.3390/antiox11020274

**Published:** 2022-01-29

**Authors:** Yijia Zhang, Zixu Wang, Yulan Dong, Jing Cao, Yaoxing Chen

**Affiliations:** Laboratory of Anatomy of Domestic Animals, College of Veterinary Medicine, China Agricultural University, Beijing 100193, China; bs20193050473@cau.edu.cn (Y.Z.); zxwang2007@163.com (Z.W.); ylbcdong@163.com (Y.D.); caojing315@126.com (J.C.)

**Keywords:** monochromatic light, oxidative stress, gut microbiota, gut development, butyrate, chick

## Abstract

Environmental light has an important impact on the growth, development and oxidative stress of chicks. Thus, we investigated the effects of colored lights on microbes and explored the molecular mechanism by which external color light information alters the gut microbiota and induces the cell response in vivo. We raised 96 chicks under 400–700 nm white (WL), 660 nm red (RL), 560 nm green (GL) or 480 nm blue light (BL) for 42 days. We used 16S rRNA high-throughput pyrosequencing and gas chromatography to explore the effect of different monochromatic lights on the jejunal microbiota. We used qRT-PCR, western blotting, immunohistochemistry and Elisa to determine the effect of different monochromatic lights on small intestine development and oxidative stress levels. With consistency in the upregulation of antioxidant enzyme ability and anti-inflammatory cytokine level, the 16S rRNA and gas chromatography results showed that BL significantly increased the diversity and richness of the jejunal microbiota and improved the relative abundances of *Faecalibacterium*, *Ruminiclostridium_9* and metabolite butyrate content compared with WL, RL and GL (*p* < 0.05). In addition, we observed that BL increased the goblet cell numbers, PCNA cell numbers, villus-length-to-crypt-depth (V/C) ratios, ZO-1, Occludin, and Claudin-1 protein expression; decreased permeability; and enhanced the digestion and absorption capacity in the jejunum (*p* < 0.05). In the in vitro experiment, we found that butyrate promoted chick small intestinal epithelial cell (CIEC) proliferation and inhibited apoptosis (*p* < 0.05). These responses were abrogated by the Gi inhibitor, PI3K inhibitor or AKT inhibitor, but were mimicked by GPR43 agonists or the GSK-3β inhibitor (*p* < 0.05). Overall, these findings suggested that BL increased the relative abundance of *Faecalibacterium*, *Ruminiclostridium_9* and butyrate production. Butyrate may act as one of the signals to mediate blue-light-induced small intestinal development and mucosal barrier integrity enhancement and promote cell proliferation via the GPR43/Gi/PI3K/AKT/p-GSK-3β/β-catenin pathway.

## 1. Introduction

In recent years, the demand for chicken meat and eggs has increased exponentially; the production of chicken meat will increase from 117 million tons to 132 million tons in 2026 [1]. Due to huge market demand, a growing number of poultry farms are making technological changes to boost production. Currently, a switch from traditional lighting sources to LED is underway. This switch will effectively decrease energy usage, reduce fear and stress, increase growth performance and improve animal welfare [2]. Chicks have highly developed visual systems, and they are highly sensitive to light [3]. Therefore, light, as an important environmental stimulus, could influence the endocrine pathways, oxidative stress level and metabolism function by disrupting the circadian rhythm of chicks [4]. In the early growth stage, the chicks raised under 560 nm green light had higher mitotic activity in their satellite cells [5] and a higher proliferation activity in their T lymphocytes [6], whereas these beneficial effects were most pronounced under 480 nm blue light in the later stage [2]. By contrast, chicks raised in 660 nm red light grew slowly, had a low level of growth hormone and high oxidative stress levels [7]. Given the many roles that green light or blue light can play in increasing the ability to adapt to the environment and upregulate production performance, the effect of different wavelengths of light in the environment on the composition of the jejunal microbiota and the mechanism underlying the manner in which external light color information induces the cell response in vivo remains to be explained.

Recently, a series of studies found that the gut microbiota is closely related to metabolite homeostasis [8], immunocompetence maintenance [9], body growth and development [10] and overall health [11], suggesting that gut microbiome homeostasis is a key indicator of health. In poultry, there are 10^9^–10^11^ cells/g bacterial harbors in the small intestine, which are dominated by *Lactobacillus*, *Enterococcus* and various *Clostridiaceae* [12]. Interestingly, the intestinal microbiota composition in chicks could be regulated by many environmental elements [13], such as temperature [14], season [15], and stocking density [16]. However, there is a gap in knowledge on the role of monochromatic lights in the jejunal microbiota’s structure and function. Therefore, our study highlights the effects of light on the microbe, reflecting the breadth of studies on light response in microbes. Besides, the research on intestinal microbiota of chicks mainly focuses on the cecum, while there is less published research on the jejunal microbiota of chicks. In addition, one hypothesis is that environmental factors cause dysregulation of gut microbes by altering the oxidative stress level and inflammatory response [17]. However, whether different wavelengths of light have an impact on chick intestinal oxidative stress levels and inflammatory responses is unclear.

Several mechanistic relationships between photoperiod and gut microbiome community have been analyzed in detail [18]. However, few studies have clarified the molecular mechanism through which monochromatic light affects intestinal microbial structure and function, especially in chicks. Butyrate, as the most representative short-chain fatty acid, is necessary for the survival of intestinal epithelial cells and can act as a cell cycle inducer to promote cell proliferation and differentiation [19,20]. Thus, whether different monochromatic lights induce intestinal epithelial cell proliferation by altering the intestinal microbial metabolite butyrate concentration should be considered.

It is crucial to understand how intestinal microbes respond to various wavelength light conditions and how to optimize the light regime to enhance chicks’ nutrient utilization rate and reduce fecal discharge. Thus, in this study, we exposed chicks to different wavelengths of monochromatic light until P42 and used multiplex sequencing of 16SrRNA gene amplicons and gas chromatography to: (1) investigate the effect of different monochromatic lights on the composition of the jejunum microflora and the development of the small intestines of chicks; (2) detect the relationship between gut health, oxidative stress and gut microbiota acquisition under four different regimens of light wavelength; and (3) delve into the molecular mechanism through which monochromatic light alters gut microbiome communities and induces the cell response in vivo. Our findings provide new theoretical support for the future use of monochromatic lighting in poultry farming and new ideas about the relationship between gut microorganisms and light.

## 2. Materials and Methods

### 2.1. Animals and Treatments

After obtaining approval from the Animal Welfare Committee of China Agricultural University, we started the experiments. The permission number was No. CAU 20171114-2.

In total, 96 post-hatching Day P0 AA male chicks were purchased from Beijing Hua du Breeding Company and raised in white, red, green or blue light under an LED system (Zhongshan Junsheng Lighting Technology Co., Ltd. Zhongshan, China) for 42 days. Therefore, there were four light treatment groups: 400–700 nm white light group (WL), 660 nm red light group (RL), 560 nm green light group (GL), and 480 nm blue light group (BL). Chicks (*n* = 24) in each light treatment group were raised in separate rooms. Each room contained three cages (eight chicks per cage) at a density of 11.5 chicks/m^2^, and each cage had independent light sources. Two LED circuit boards were installed at the top of each side of each cage, and each circuit board had eight LED lamps. The distance between each lamp was 6 cm. The light intensity was around 0.19 W/m^2^ and the light regime was 23 light: 1 dark (light on at 00:00 and off at 23:00).

In the first week, the temperature in each room was kept at 32 ± 2 °C; subsequently, it was reduced by 1 °C every two days, until the temperature in each room reached 30 °C. The relative humidity of each room was 60%. The chicks enjoyed food and drink ad libitum. The basal diet nutrient compositions are shown in Table 1. The nutrient level of this basal diet met the nutrient recommendations of the National Research Council for chicks (1994).

### 2.2. Sampling

At P42, all chicks were intraperitoneally injected with pentobarbital sodium (30–40 μg/g) and decapitated after anesthesia. Their jejunal tissue and content were then harvested. Four chicks were randomly selected from WL, RL, GL and BL, and their jejunal content was collected for microbial sequencing. In addition, six chicks from WL, RL, GL and BL were used for inflammatory factors, antioxidant capacity and intestinal permeability assay. The fresh jejunal content was collected for volatile fatty acid (VFA) analysis. Another five chicks from each of the remaining groups were selected and the jejunum of each was collected for immunohistochemistry, western blot and qRT-PCR assay.

### 2.3. Chick Normal Small Intestinal Epithelial Cells Culture and Treatment

CIECs were (ATCC Cell Bank, Shanghai, China) prepared with DMEM, including 10% fetal bovine serum (FBS) and 1% antibiotics. Next, different butyrate concentrations (0, 0.1, 0.5, 2 or 5 mM; Sigma-Aldrich, St. Louis, MO, USA) were added into the medium and incubated at 37 °C in 5% CO_2_ for 24 h. The proliferation level of the CIECs was determined by methyl thiazolyl tetrazolium (MTT) assay. We used the stimulation index (SI) to express the proliferative activity of CIECs. SI = OD570 (stimulated cells)/OD570 (unstimulated cells).

In addition, to explore the intracellular signaling pathway activated by butyrate, we added 10 μM LY294002 (an inhibitor of PI3k, MCE, Weehawken, NJ, USA), 2 μM GSK690693 (an inhibitor of AKT, MCE, Weehawken, NJ, USA), 5 μM CHIR99021 (an inhibitor of GSK-3β, MCE, Weehawken, NJ, USA), 20 μM 4-CMTB (an agonist of GPR43, MCE, Weehawken, NJ, USA), 5 μM AR420626 (an agonist of GPR41, GlpBio, Montclair, NJ, USA), 50 ng/mL PTX (an inhibitor of Gi, List Biological Laboratories, Campbell, CA, USA) and 10 μM Ym254890 (an inhibitor of Gq, Wako Pure Chemical Industries, Ltd. Osaka, Japan), respectively, into CIECs for 0.5 h before the addition of exogenous butyrate (0.5 mM, Sigma-Aldrich, St. Louis, MO, USA). Twenty-four hours later, CIECs were collected for western blot assay. Each assay used a repeat of six wells.

### 2.4. Antioxidant Parameters Measurements

The jejunums (*n* = 6) were collected for antioxidant activity analysis. The SOD, CAT, GSH-Px, T-AOC and MDA analyses were performed by using commercial kits (Beyotime Co., Ltd., Shanghai, China). The experimental protocol was completed according to the instructions. Each sample was tested three times.

The ROS level of each jejunum was detected by using a commercial kit (Nanjing Jiangcheng Co., Ltd., Nanjing, China). Briefly, the jejunal tissues (*n* = 6) were washed using PBS, sheared and made into single-cell suspensions (1 × 10^5^ cells/mL). Next, 10 μm 2′-7′-dichlorofluorescein diacetate (DCFH-DA) was used to incubate cells for half an hour at 37 °C in the dark. The fluorescence intensity was determined by a microplate reader (502 nm excitation and 530 nm emission, Synergy HT; BioTek, Winooski, VT, USA). The fluorescence intensity per mg of jejunum was calculated as the percentage ROS compared to control for jejunal tissue. Each sample was tested three times.

### 2.5. Enzyme-Linked Immunosorbent Assay

Jejunal IL-10, IL-6, TNF-α and IFN-γ levels were determined by ELISA (Uscn Life Science, Inc., Wuhan, China). The experimental program was completed according to the manufacturer’s guidance (intra-assay CV = 7.9%). Each sample was tested in triplicate.

### 2.6. Intestinal Permeability

Briefly, at 42 d, one chick from each light treatment group was given a gavage of 1 mL fluorescein isothiocyanate dextran solution (FITC-d; 2.2 mg/mL; MW 4000; Sigma, Adrich, St. Louis, MO, USA). The chicks were euthanized 2 h later and blood was collected from their veins. After centrifugation at 1000× *g* for 15 min, the serum was collected. Next, the FITC-d concentration per mL of serum was determined by a microplate reader (485 nm excitation and 535 nm emission, Synergy HT; BioTek, Winooski, USA). In addition, PBS was used to dissolve FITC-d to make the standard curve and the FITC-d concentration per mL of serum was determined according to the standard curve.

### 2.7. Histological Staining and Immunohistochemical Staining

The freshly removed jejunal tissues (*n* = 5) were quickly fixed in 4% paraformaldehyde for 48 h. Next, the tissues were dehydrated, embedded and finally made into 5 μm thick paraffin sections. These tissue sections were then dewaxed in xylene and dehydrated in descending grades of alcohol. Subsequently, these paraffin sections were stained using hematoxylin-eosin (H&E) to measure the villus length (V) and crypt depth (C). We randomly selected sixty fields in six sections of each sample and photographed them with a 400 objective on a microscope (BX51, Olympus, Tokyo, Japan). From each field, we selected the five longest villi and the 300 longest villi per sample were analyzed. In addition, we used periodic acid-Schiff [21] to measure the number of goblet cells per 100 absorbed cells. At least 30 fields in 6 sections of each sample and a total of 150 fields per group were photographed. Next, the number of goblet cells per 100 absorbed cells was calculated.

For immunohistochemical staining, the primary antibodies (rabbit anti-PCNA, 1:500; rabbit anti-MUC2, 1:180; Abcam, Cambridge, UK) were incubated with the sections overnight at 4 °C. Next, these sections were incubated with secondary antibody (biotinylated goat anti-rabbit IgG, 1:200, Sigma, St. Louis, MO, USA) for 2 h at 25 °C. After washing in PBS, these sections were incubated with streptavidin-horseradish peroxidase (1:200, Sigma, St. Louis, MO, USA) for 2 h at 25 °C and visualized by incubating 0.05% DAB (Sigma, St. Louis, MO, USA) and 0.003% H_2_O_2_. Positive cells in five cross-sections were randomly selected for each sample, and at least twenty-five fields were counted. We incubated sections in the negative control group with PBS instead of primary antibody and other steps remained unchanged. We used Image-Pro Plus software (Media Cybernetics, Inc., Rockville, MD, USA) to integrate optical density (IOD).

### 2.8. Real-Time Reverse Transcription-Polymerase Chain Reaction (qRT-PCR)

A TRIzol reagent (CoWin Biotech Co., Inc., Beijing, China) was used to extract jejunal total RNA (*n* = 5) and a cDNA synthesis kit (Thermo Fisher, Boston, MA, USA) was used to synthesize cDNA. Next, using the AceQ qPCR SYBR green master mix (Vazyme Biotech, Nanjing, China), we proceeded to RT-PCR amplification. The primers involved in this research are shown in Table 2 and each sample was repeated in triplicate. The relative mRNA level was normalized to the GAPDH gene expression level.

### 2.9. Western Blot Analysis

A RIPA lysis buffer (Beyotime Co., Ltd., Shanghai, China) was used to extract jejunal protein (*n* = 5) and a BCA kit (Beyotime Co., Ltd., Shanghai, China) was used to determine the protein concentration. We then added 20 μL protein from each sample into the SDS-PAGE before transferring onto PVDF membranes. Next, we used 5% skimmed milk to blocked nitrocellulose membranes for 1 h. Subsequently, the primary antibodies, including anti-Claudin-1 (rabbit, 1:1000; Invitrogen, Carlsbad, CA, USA), anti-Occludin (rabbit, 1:1000;, Invitrogen, Carlsbad, CA, USA), anti-ZO-1 (rabbit, 1:1000; Invitrogen, Carlsbad, CA, USA), anti-phospho-PI3 Kinase (Tyr458) antibody (rabbit, 1:1000, CST, Boston, MA, USA), anti-phosphor-AKT (Ser473) antibody (rabbit, 1:500, CST, Boston, MA, USA), anti-AKT antibody (rabbit, 1:500, CST, Boston, MA, USA), anti-phosphor-GSK-3β antibody (rabbit, 1:500, Abnova, Taiwan, China), anti-GSK-3β antibody (rabbit, 1: 500, Proteintech Group, Inc, Wuhan, China), anti-β-catenin antibody (rabbit, 1:1000, Proteintech Group, Inc, Wuhan, China), anti-cyclinD1 antibody (mouse, 1:200, Abbexa, Cambridge, UK), anti-Bax antibody (goat, 1:1000, Biorbyt, NJ, USA), anti-Bcl-2 antibody (rabbit, 1:1000, Biorbyt, NJ, USA), anti-Caspase-3 antibody (rabbit, 1:1000, CST, Boston, MA, USA) and anti-β-actin (mouse, 1:4000; Co Win Biotech Co., Inc, Beijing, China), were incubated with the membranes overnight at 4 °C. Next, nitrocellulose membranes were incubated with HRP-conjugated goat anti-mouse/rabbit antibody (1:8000; Co Win Biotech Co., Inc. Beijing, China) for 2 h. The IOD of the target bands was measured by using ImageJ software (version 4.0.2; Scion Corp., Frederick, MD, USA) and normalized to the corresponding β-actin values. Each sample was tested in triplicate.

### 2.10. Microbial Sequencing and Analysis

The total DNA (*n* = 4) of each jejunum was extracted with a QIAamp DNA Stool Mini Kit (Hilden, Germany). The V3-V4 region of the 16S rRNA gene was amplified by using PCR and the detailed method was modified as previously described [22]. The sequencing data we generated were deposited in the NCBI Sequence Read Archive (SRA) under accession numbers from SAMN19812657 to SAMN19812672 in PRJNA739905.

According to UCLUST, the effective reads of each sample were clustered into operational taxa with 97% sequence similarity. Next, we used QIIME for RDA identification of key OTUs. β-diversity was estimated by calculating weighted UniFrac distance, visualized using principal coordinate analysis (PCoA) and plotted using the “Vegan” and “GGplot2” software packages in R software (Version 3.4.4, Vienna, Austria). PERMANOVA (similarity analysis) was used to evaluate the significance of microbial structural differentiation between four groups. R package was “pure”. Relative abundances of all differential phyla and genera (*p* < 0.05) in each group were analyzed by using the nonparametric factorial Kruskal-Wallis H test. The dominant pathway difference between groups was detected using Kyoto encyclopedia of genes and genomes (KEGG) difference analysis. Functional genes were predicted using PICRUSt (phylogenetic investigation of communities by reconstruction of unobserved states) using high-quality sequences as the input.

### 2.11. SCFAs Extraction and Analysis

For short-chain fatty acid (SCFAs) analysis, we collected fresh jejunal content (*n* = 6) samples and diluted them 5 fold with PBS. Next, we centrifuged the diluted jejunal content at 4731× *g* for ten mins at 4 °C and mixed 1 mL supernatant with 200 μL metaphosphoric acid solution. After soaking in ice water for 30 min, the content was centrifuged at 5595× *g* for ten mins at 4 °C. Next, the gas chromatography (Agilent 6890N, Agilent Technologies, Inc., Beijing, China) was filled with supernatant to determine the concentrations of acetate, propionate and butyrate. Finally, the concentration was calculated by using the raw data multiplied by the dilution ratio.

### 2.12. Statistical Analysis

The values of each group were shown as the mean ± standard error of the mean (SEM). Before analyses, we used the Kolmogorov-Smirnov test to assess whether the data were normally distributed. Except for sequencing data, the other variables met the assumptions for normality. Next, we used one-way ANOVA to evaluate the significant difference between four different light treatment groups by using SPSS 25.0 software (SPSS, Chicago, IL, USA). Correlation analysis, expressed as Spearman’s correlations (r > 0.5, *p* < 0.05), was performed to determine the correlations between the abundance of microbiota and intestinal development.

## 3. Results

### 3.1. Effect of Various Monochromatic Lights on Altered Gut Microbiota Composition

**Figure 1:**
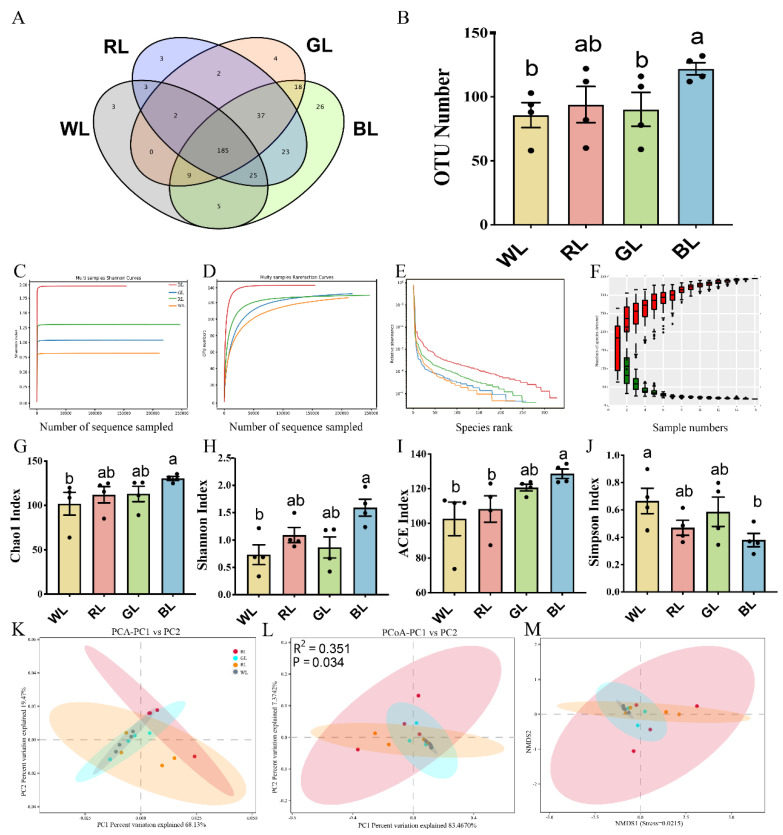
Effects of different monochromatic lights on the OTU number (**A**,**B**), Shannon curves (**C**), rarefaction curves (**D**), OTU rank curves (**E**), rank abundance curve (**F**), Chao1 index (**G**), Shannon index (**H**), ACE index (**I**), Simpson index (**J**), principal component analysis (PCA) (**K**), PCoA score plot (**L**), nonmetric multidimensional scaling (NMDS) score plot based on the weight UniFrac score plot based on the OTU (**M**) of the gut microbiota in the jejunum of WL, RL, GL and BL at P42. WL: white light; RL: red light; GL: green light; BL: blue light. These results are shown as means ± SEM. Differences between the four groups are presented in the form of different letters (*p* < 0.05).

Next, we assessed whether different monochromatic lights could influence the jejunal microbiota composition in chicks at P42. According to the 16S rRNA sequencing, 16 samples, 266,022, 297,710, 269,625 and 196,981 raw reads and 55,009, 65,229, 58,298 and 41,918 clean tags were obtained from WL, RL, GL and BL (*n* = 4), respectively. As shown in Figure 1A, we identified 346 OTUs, 185 of which appeared to be present in all the samples, whereas 3 unique OTUs (OTU216, OTU761, OTU283) belonged to the phyla of *Firmicutes* and *Proteobacteria* in WL; 3 unique OTUs (OTU180, OTU235, OTU940) belonged to the phylum *Firmicutes* in RL; 4 unique OTUs (OTU195, OTU226, OTU240, OTU302) belonged to the phylum *Firmicutes* in GL; and 26 unique OTUs belonged to the phylum *Firmicutes* in BL. As shown in Figure 1B, the OTU number of BL was 29.79–42.27% higher than in WL, RL and GL. By comparing the rarefaction curve and Shannon curves between the four groups, we found that the new OTUs and Shannon index declined with the increase in the sequencing number, which indicated that our samples covered most microbial species information (Figure 1C–F). Furthermore, we used an alpha diversity analysis to determine the species richness and the diversity of the microbial communities. As shown in Figure 1G–J, BL significantly upregulated the Chao1 (*p* = 0.046), Shannon (*p* = 0.035) and Ace (*p* = 0.002) indices while significantly decreasing the Simpson index compared with WL (*p* = 0.027). In addition, there was a significant difference between BL and RL with regards to the ACE index (*p* = 0.014).

Next, the β-diversity assays, including the PCA, PCoA and NMDS assays, were used to determine the degree of dispersion of the microorganisms between the different light treatment groups. PCA and PCoA based on weight UniFrac distance in the jejunum showed that the samples from WL, RL, GL and BL were separated (Figure 1K,L). These microbial community profiles from the four groups were significantly different according to PERMANOVA (R^2^ = 0.351, *p* = 0.034). A similar result was observed in the nonmetric multidimensional scaling (NMDS) assay (Figure 1M). The alpha diversity index and β-diversity index demonstrated that WL was quite different from the other three groups in terms of the structural composition of the jejunal intestinal microbiota.

### 3.2. Abundance and Significant Difference between Four Groups at the Phylum Level

**Figure 2:**
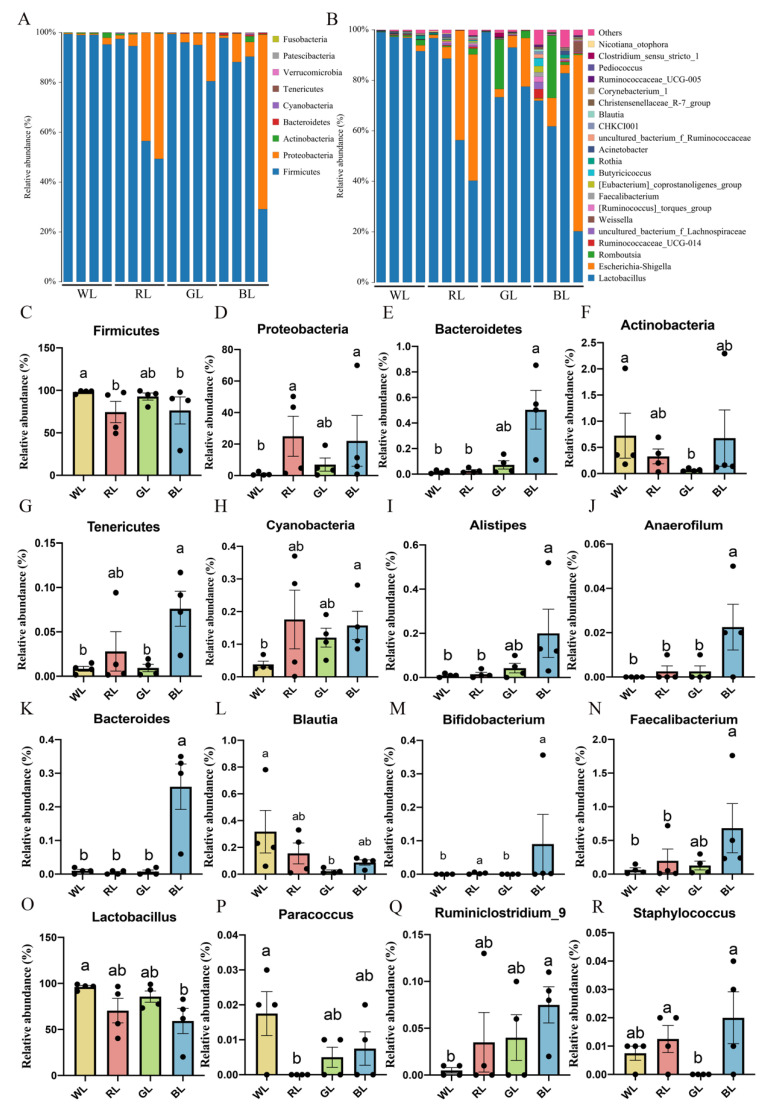
The relative contribution of the top 10 phyla in the WL, RL, GL and BL (**A**). Twenty-two genera were identified that were present at a relative abundance of >0.1% in the WL, RL, GL and BL (**B**). Taxonomic profiles of the notably significantly different bacteria at the phylum level (**C**–**H**) and genus level (**I**–**R**). WL: white light; RL: red light; GL: green light; BL: blue light. These results are shown as means ± SEM. Differences between the four groups are presented in the form of different letters (*p* < 0.05).

As shown in Figure 2A, we found that the dominant bacteria in the jejunum were *Firmicutes* (WL: 98.20%, RL: 74.50%, GL: 92.70% BL: 76.40%) and *Proteobacteria* (WL: 1.02%, RL: 25.00%, GL: 6.98% and BL: 22.00%) (Figure 2A), while *Bacteroidetes* and *Actinobacteria* manifested smaller percentages in the chick jejunum gut at the phylum level. As shown in Figure 2C, the relative abundance of *Firmicutes* in WL was higher than in RL and BL (*p* = 0.031–0.038). Additionally, as shown in Figure 2D, the relative abundance of *Proteobacteria* was higher in RL and BL than in WL (*p* = 0.026–0.031). *Bacteroidetes* (*p* = 0.006), *Tenericutes* (*p* = 0.026), and *Cyanobacteria* (*p* = 0.038) were more abundant in BL than in WL (Figure 2E,G,H). The abundance of *Actinobacteria* in GL was significantly higher than in WL (*p* = 0.014) (Figure 2F). These results demonstrated that each light treatment group had its unique feature distribution at the phylum level.

### 3.3. Abundance and Significant Differences between the Four Groups at the Genus Level

Twenty-two genera were identified that presented a relative abundance of >0.1%, and *Lactobacillus*, *Escherichia-Shigella*, and *Romboutsia* accounted for >98% of the sequences (Figure 2B). The relative abundances of *Alistipes* (Figure 2I), *Anaerofilum* (Figure 2J), *Bacteroides* (Figure 2K), *Blautia* (Figure 2L), *Bifidobacterium* (Figure 2M), *Faecalibacterium* (Figure 2N), and *Ruminiclostridium_9* (Figure 2Q) were significantly increased (*p* < 0.05), and the relative abundance of *Lactobacillus* (Figure 2O) was significantly decreased in BL compared with WL.

### 3.4. Differences in Predicted Functional Properties between the Four Groups

**Figure 3:**
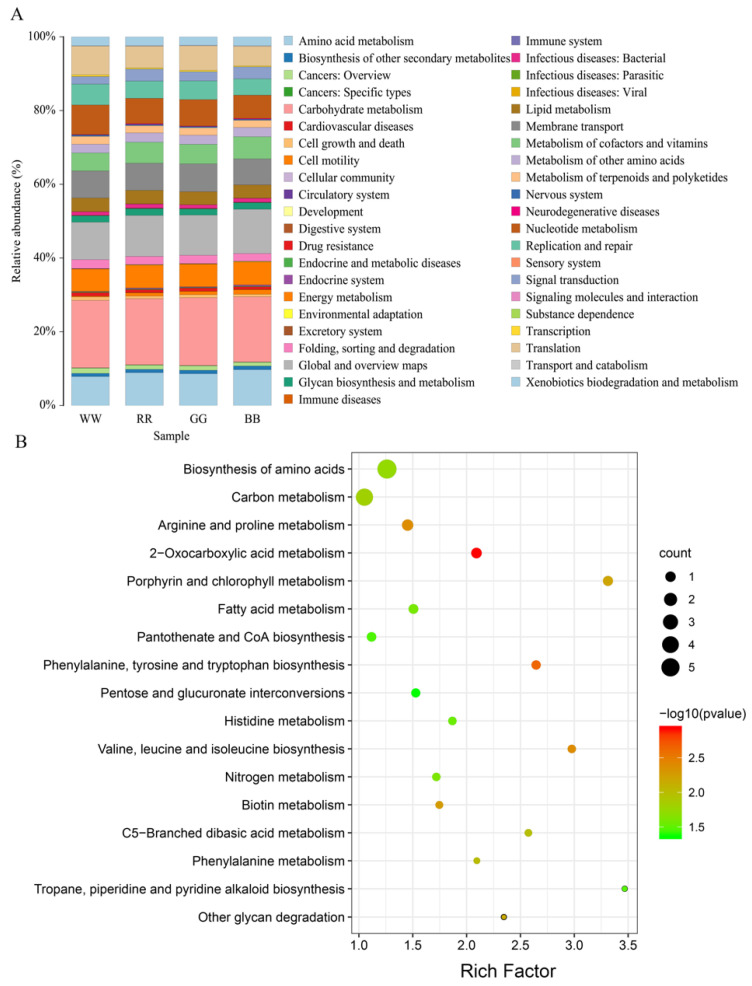
KEGG difference analysis of WL, RL, GL and BL (**A**). Scatter plot of enriched KEGG pathways statistics among WL and BL (**B**). On the KEGG enrichment scatter plot, the 17 most significant enriched pathways in WL and BL are presented. The y-axis represents the name of the pathway and the x-axis represents the rich factor. The rich factor is the ratio of the differentially expressed gene number in BL to WL in a certain pathway. The count size stands for the number of different genes and the color stands for different *p*-values. WL: white light; RL: red light; GL: green light; BL: blue light.

Next, we aimed to explore the difference in the predicted functional properties based on the 16sRNA between the four light treatment groups. The presumptive functions of the jejunal microbiota were illustrated using PICRUSt. Compared to the taxonomic profiles, the functional profiles of the four groups were more similar. Carbohydrate metabolism (18.45%), global and overview maps (10.01%), amino acid metabolism (7.71%), membrane transport (7.51%), nucleotide metabolism (8.05%), translation (7.83%), energy metabolism (6.08%), the metabolism of cofactors and vitamins (4.79%), the replication and repair (5.66%) and lipid metabolism (3.70%) were identified as the top 10 predicted functions for the jejunal microbiota in WL, they were also the top 10 predicted functions in RL, GL and BL (Figure 3A). As shown in Figure 3B, there were a total of 17 different metabolic pathways in level 3 KEGG identified between WL and BL. Many of these metabolic pathways were enriched in amino acids, carbohydrates and energy metabolism. There were also a few metabolic pathways involved in cofactor and vitamin metabolism, global and overview maps and the immune system.

### 3.5. Effect of Various Monochromatic Lights on Jejunal Butyrate Concentrations and Membrane Receptor GPR41 and GPR43 mRNA Expression in the Jejunum

**Figure 4:**
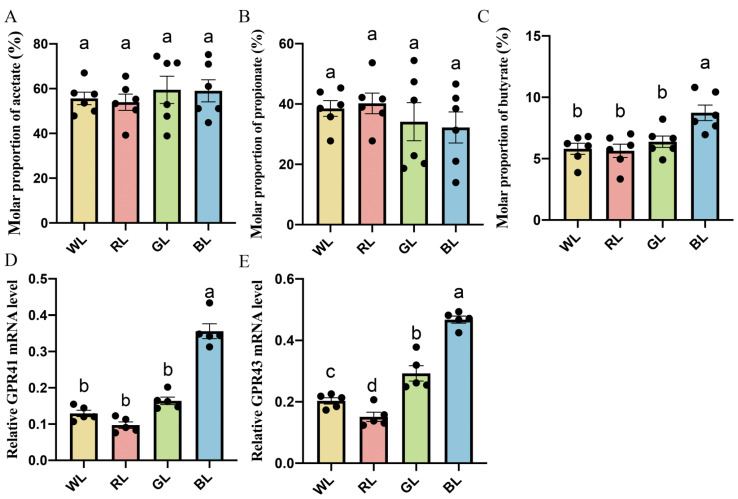
Effects of different monochromatic lights on the jejunal acetate concentration (**A**), jejunal propionate concentrations (**B**), jejunal butyrate concentration (**C**), *GPR41* mRNA expression (**D**) and *GPR43* mRNA expression (**E**) in the jejunums of WL, RL, GL and BL at P42. WL: white light; RL: red light; GL: green light; BL: blue light. These results are shown as means ± SEM. Differences between four groups are presented in the form of different letter (*p* < 0.05).

As shown in Figure 4A,B, the gas chromatography results showed that there was no significant difference in the content of acetate and propionate concentration between WL, RL, GL and BL. However, the chicks exposed to BL showed a significantly higher content of butyrate than the chicks reared under WL, RL and GL by 36.99–50.43% (*p* = 0.001–0.006) at P42. In addition, as shown in Figure 4D,E, BL significantly upregulated *GPR43* and *GPR41* mRNA expression by 59.72–208.96% (*p* = 0.001) and 115.89–279.26% (*p* = 0.001) compared with WL, RL and GL, respectively.

### 3.6. Effects of Different Monochromatic Lights on Jejunal Cyclin d1, Caspase-3, Bcl-2 and Bax Protein Expression

**Figure 5:**
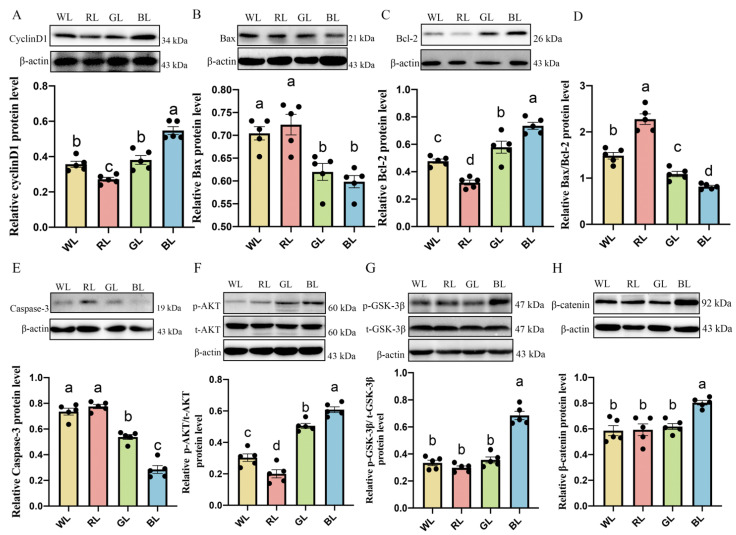
Effects of different monochromatic lights on chick jejunum cyclinD1 protein (**A**), Bax protein (**B**), Bcl-2 protein (**C**), Bax/Bcl-2 ratio (**D**), Caspase-3 protein (**E**) p-AKT/total-AKT ratio (**F**), p-GSK-3β/total-GSK-3β ratio (**G**), and β-catenin protein level (**H**) in the jejunums of WL, RL, GL and BL at P42. WL: white light; RL: red light; GL: green light; BL: blue light. These results are shown as means ± SEM. Differences between four groups are presented in the form of different letters (*p* < 0.05).

As shown in Figure 5A,C, BL remarkably promoted the protein expression of cyclin D1 (*p* < 0.001) and Bcl-2 (*p* < 0.001), whereas the protein of Bax (Figure 5B, *p* < 0.001), Caspase-3 (Figure 5D, *p* < 0.001) and the ratio of Bax/Bcl-2 (Figure 5E, *p* = 0.000–0.021) were obviously lower in BL than in WL, RL and GL, respectively.

### 3.7. Effects of Various Monochromatic Lights on the Expression of p-AKT, p-GSK-3β and β-Catenin Protein in the Jejunum

To determine the intracellular signaling pathway involved in the proliferation and inhibition of apoptosis in BL-induced chick small intestinal epithelial cells (CIECs), we tested the PI3K/AKT signaling-related protein expression in the chick jejunums under WL, RL, GL and BL. As shown in Figure 5F–H, BL significantly improved the p-AKT, p-GSK-3β and β-catenin protein compared with WL, RL and GL by 20.47–204.20% (p-AKT, *p* = 0.001–0.004), 92.29–129.82% (p-GSK-3β, *p* < 0.001) and 30.05–37.15% (β-catenin, *p* < 0.001), respectively.

### 3.8. Effects of Different Monochromatic Lights on the Antioxidant Capacity of the Jejunum

**Figure 6:**
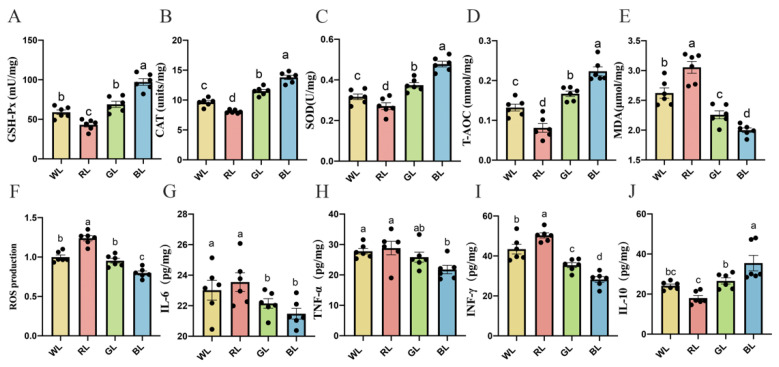
Effect of different monochromatic lights on GSH-Px (**A**), CAT (**B**), SOD (**C**), T-AOC (**D**), MDA (**E**), ROS (**F**), IL-6 (**G**), TNF-α (**H**), IFN-γ (**I**) and IL-10 (**J**) concentrations in the jejunums of WL, RL, GL and BL at P42. WL: white light; RL: red light; GL: green light; BL: blue light. These results are shown as means ± SEM. Differences between the four groups are presented in the form of different letters (*p* < 0.05).

As shown in Figure 6A–D, BL significantly improved the antioxidant enzymes and T-AOC, compared with WL, RL and GL, by 19.93–70.86% (CAT, *p* < 0.001), 41.01–125.78% (GSH-Px, *p* < 0.001), 27.77–76.31% (SOD, *p* < 0.001) and 31.92–171.36% (T-AOC, *p* = 0.001) in the jejunum. However, the MDA and ROS content, which could aggravate oxidative stress levels, were significantly decreased in BL and significantly increased in RL (Figure 6E,F).

### 3.9. Effects of Different Monochromatic Lights on Jejunal Cytokine Levels

As shown in Figure 6G–I, BL caused a decrease in jejunal pro-inflammatory cytokine IL-6 (3.03–8.79%, *p* = 0.009–0.043), TNF-α (15.79–24.50%, *p* = 0.006–0.015) and IFN-γ (20.19–43.84%, *p* = 0.001–0.009) levels. However, BL caused an increase in anti-inflammatory cytokine IL-10 (33.48-96.75%, *p* = 0.001–0.013) levels compared with WL, RL and GL, respectively (Figure 6J). By contrast, the proinflammatory cytokine IL-6, TNF-α and IFN-γ levels in RL were the highest among the other three groups.

### 3.10. Effects of Different monochromatic Lights on Jejunal Development and Jejunal Mucosa Function in Chicks

**Figure 7:**
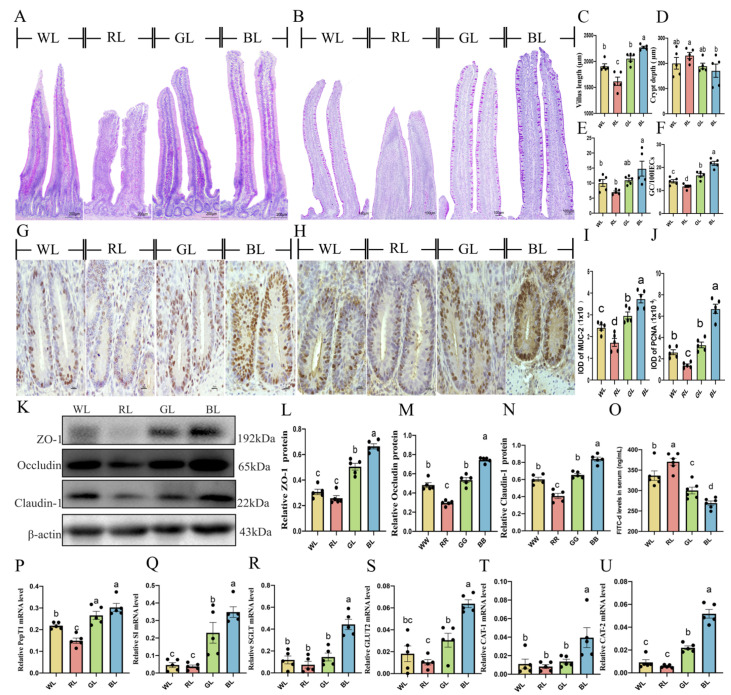
Effects of different monochromatic lights on HE staining of jejunal tissue sections (**A**), PAS staining of jejunal tissue sections (**B**), jejunal villus height (scale bar = 100 μm) (**C**), jejunal crypt depth (**D**), jejunal villus height/crypt depth (V/C) ratio (**E**), goblet cell numbers (scale: 100 μm) (**F**), immunohistochemical staining photographs of MUC-2 (scale: 20 μm) (**G**), immunohistochemical staining photographs of PCNA (scale: 20 μm) (**H**), IOD of MUC2-positive cells (**I**), IOD of PCNA-positive cells (**J**), ZO-1, Claudin-1 and Occludin protein expression (**K**–**N**), intestinal permeability (**O**), *PepT1* mRNA level (**P**), *SI mRNA* level (**Q**), *SGLT1* mRNA level (**R**), *GLUT2* mRNA level (**S**), *CAT-1* mRNA level (**T**), *CAT-2* mRNA level (**U**) in chicks of WL, RL, GL and BL at P42. WL: white light; RL: red light; GL: green light; BL: blue light. These results are shown as means ± SEM. Differences between the four groups are presented in the form of different letters (*p* < 0.05). PepT1, influx oligopeptide transporter peptide transporter 1; SI, sucrose-isomaltase; SGLT1, Na^+^-glucose cotransporter; GLUT2, glucose transporter type 2; CAT1, transporter 1 of cationic amino acid; CAT2, transporter 2 of cationic amino acid.

We examined the morphological changes and development of intestinal villi in the jejunum. As shown in Figure 7A, BL significantly increased the length of the jejunal villus and decreased the crypt depth, which resulted in an increase in the V/C ratio compared with WL, RL and GL (34.67–110.39%, *p* = 0.001–0.075). As shown in Figure 7B, the number of goblet cells per 100 absorbed cells in the BL was increased by 29.00-81.69% (*p* = 0.000) compared with those in WL, RL and GL. Similar results were observed in the IOD of MUC2 and PCNA (Figure 7G–J). The western blot results also showed that ZO-1, Occludin and Claudin-1 protein were markedly upregulated, by 10.73–158.33% (Figure 7K–N, *p* = 0.000–0.001), in the BL compared with the WL, RL and GL. Furthermore, the concentration of FITC-D in serum was lowest in BL and highest in RL (Figure 7O, *p* = 0.000–0.020). Therefore, these results indicated that red light had the most severe gastrointestinal permeability and blue light had a promoting effect on small intestine development and mucosal integrity.

Next, we explored whether various monochromatic lights have an impact on the digestion and absorption of poultry. Thus, we used q-PCR to examine the mRNA expression of *intestinal influx oligopeptide transporter peptide transporter 1 (PepT1)*, *sucrose-isomaltase*, *Na+-glucose cotransporter (SGLT1)*, *glucose transporter type 2 (GLUT2)* and *cationic amino acid transporter 1,2 (CAT1, CAT2)*. As shown in Figure 7Q–U, BL significantly increased by 70.98–915.63% (*SI*, *p* = 0.000–0.025), 280.68–610.16% (*SGLT1*, *p* = 0.000), 98.68–481.11% (*GLUT2*, *p* = 0.008–0.001), 181.46–386.23% (*CAT1*, *p* = 0.015–0.040) and 140.56–785.47% (*CAT2*, *p* = 0.000) in the jejunums of chicks versus WL, RL and GL. In addition, as shown in Figure 7P, the *PepT1* mRNA expression in BL was higher than in RL and WL by 38.46–103.17% (*p* = 0.001). Overall, these results suggested that BL effectively promotes chick jejunum development, strengthens the intestinal mucosa barrier and enhances digestion and absorption.

### 3.11. Correlation Analysis between Gut Health, Oxidative Stress Level and Jejunal Microbiota

**Figure 8:**
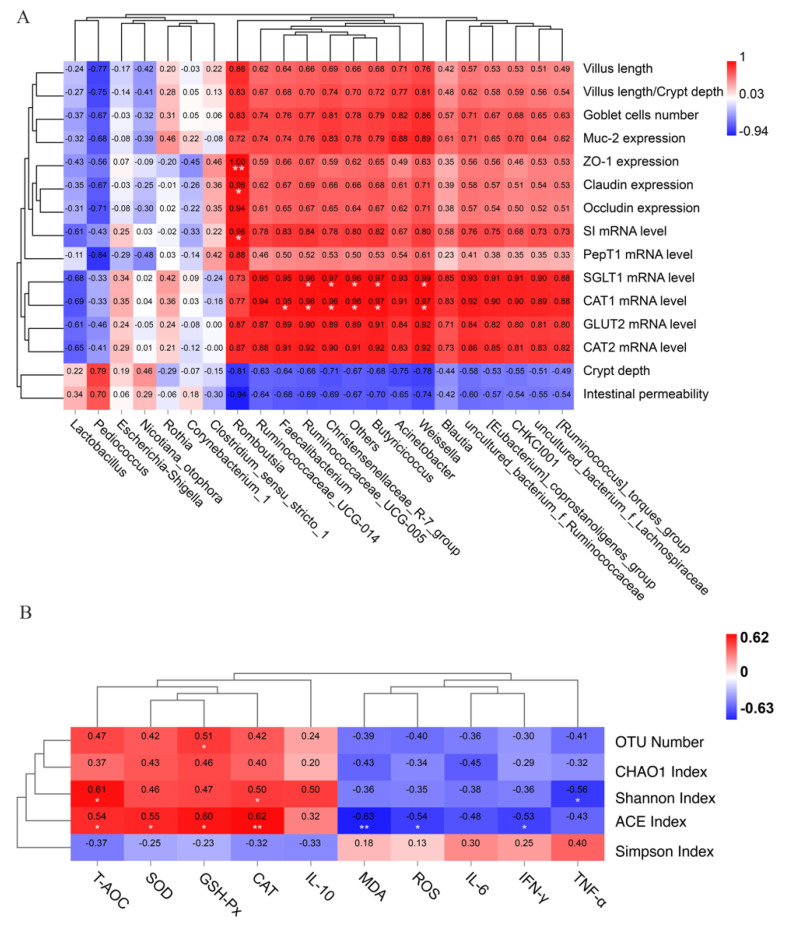
Spearman’s rank correlations between the relative abundances of 22 key phylotypes at the genus level on d 42 and gut health (**A**). Spearman’s rank correlations between the microbial composition and oxidative stress level (**B**). The colors range from blue (negative correlations) to red (positive correlations). * *p* < 0.05; ** *p* < 0.01.

To investigate specific bacteria related to gut health (including morphological integrity and physiological functions of the intestinal tract), we performed Spearman’s correlations analysis between the abundance of microbiota and intestinal development. As shown in Figure 8A, there was a significant positive correlation between intestinal barrier functions (higher ZO-1 and claudin expression,) and *Romboutsia* (r = 0.96–1.00, *p* = 0.003–0.040). Significant positive correlations between *intestinal digestive enzyme SI* and *Romboutsia* were also determined from the heatmap (r = 0.96, *p* = 0.040). In addition, the abundance of the genera *Weissella, Faecalibacterium, Butyricicoccus, Christensenellaceae_R–7_group* and *Ruminococcaceae_UCG-005* showed highly positive correlations with the *cationic amino acid transporter CAT1* and *Na+-glucose cotransporter (SGLT1)* (r = 0.095–0.099, *p* = 0.049–0.014).

In addition, we used Spearman’s correlation analysis to detect the relationship between microbial composition (including OTU numbers, ACE index, chao1 index, Shannon index, Simpson index) and oxidative stress level. As shown in Figure 8B, we found that the ACE index was positively correlated with T-AOC, SOD, CAT and GSH-Px (r = 0.062–0.054, *p* < 0.01), whereas it was negatively correlated with MDA, IL-6 and IFN-γ (r = −0.063–−0.054, *p* < 0.01). In addition, the Shannon index also showed significantly positive correlations with CAT and T-AOC (r = 0.050–0.061, *p* < 0.05) and showed a negative correlation with TNF-α (r = −0.056, *p* < 0.05).

### 3.12. Butyrate Modulates BL-Induced CIECs Proliferation in Chicks via the GPR43/Gi/PI3K/AKT/GSK-3β/β-Catenin Pathways

To affirm our hypothesis as to whether butyrate promoted the proliferation of chick small intestinal epithelial cells (CIECs), we determined the effect of 0.2–5 mM butyrate on CIECs proliferation by using an MTT assay. We used the CIEC stimulation index to express the MTT results. In this study, we found that when the concentration of exogenous butyrate was ≤0.5 mM, it effectively promoted the proliferation of CIECs. Thus, we chose 0.5 mM butyrate for the follow-up experiments.

**Figure 9:**
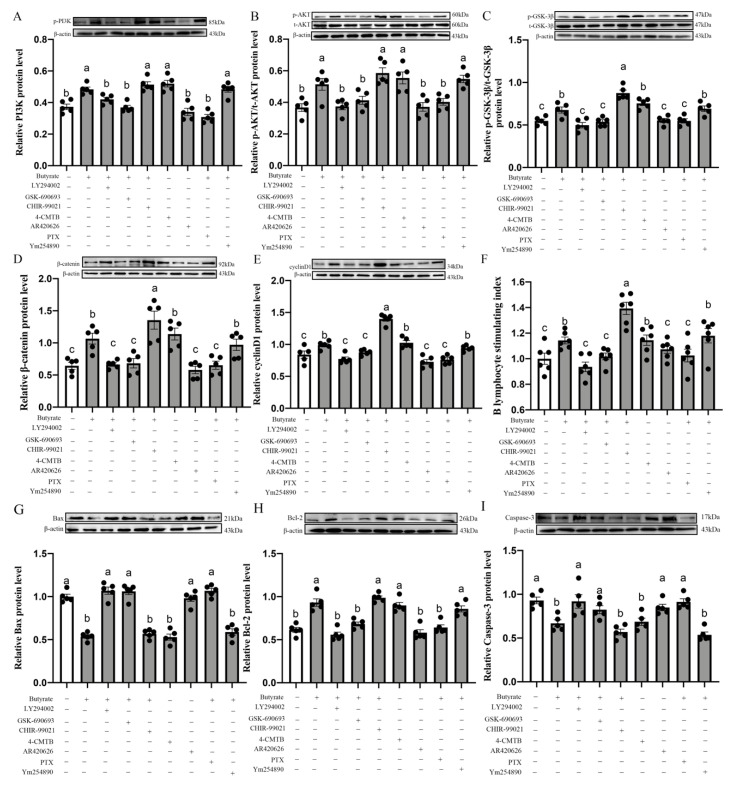
Effects of PI3K inhibitor, AKT inhibitor, GSK-3β inhibitor, GPR43 agonist, GPR41 agonist, Gi inhibitor and Gq inhibitor on p-PI3K protein expression (**A**), p-AKT/total-AKT ratio (**B**), p-GSK-3β/total-GSK-3β ratio (**C**), β-catenin protein expression (**D**), cyclin D1 protein expression (**E**) and CIECs proliferation stimulation index (**F**), Bax protein expression (**G**), Bcl-2 protein expression (**H**) and Caspase-3 protein expression (**I**). LY294002 is an inhibitor of PI3K; GSK-690693 is an inhibitor of AKT; CHIR-99021 is an inhibitor of GSK-3β; 4-CMTB is an agonist of GSK-3β; AR420626 is an agonist of GPR41; PTX is an inhibitor of Gi and Ym254890 is an inhibitor of Gq. Different letters show significant differences among the various treatment groups (*p* < 0.05). p-PI3K: phosphorylated PI3-kinase; p-AKT: phosphorylated AKT; p-GSK-3β: phosphorylated GSK-3β. CIECs: chick small intestinal epithelial cells.

Consistent with the changes in cell proliferation activity in the CIECs, we observed an upregulation of PI3K (29.67%, *p* = 0.001; Figure 9A), p-AKT (40.03%, *p* = 0.002; Figure 9B), p-GSK-3β (23.52%, *p* = 0.003; Figure 9C), β-catenin (65.68%, *p* = 0.001; Figure 9D) and cyclinD1 (17.54%, *p* = 0.004; Figure 9E) protein expression in the butyrate group compared with the control group. Furthermore, the pretreatment of the CIECs with 4-CMTB (GPR43 agonist) had similar effects. However, the pretreatment of the CIECs with AR420626, which was an agonist of GPR41, showed no significant effect on the CIECs stimulation index and cyclin D1 protein expression. These results indicated that butyrate may promote blue-light-induced CIEC proliferation mediated by GPR43, but not by GPR41.

In addition, we used Gi inhibitor PTX or Gq inhibitor Ym254890 to determine which G proteins were bound to GPR43 for transmitting butyrate signals to the CIECs. Through western blot and MTT assays, we found that PTX significantly reduced the expression of cyclinD1 protein and the stimulation index of CIECs, respectively, compared with the butyrate group, (Figure 9E,F). However, Ym254890 did not have this inhibitory effect (*p* = 0.332–0.531). Similarly, LY294002 (PI3K inhibitor) and GSK-690693 (AKT inhibitor) significantly abrogated the butyrate-induced upregulation of p-PI3K, β-catenin and cyclinD1 protein, decreased the p-AKT/total-AKT and p-GSK-3β/total-GSK-3β ratio and inhibited CIEC proliferation. By contrast, the pretreatment of CIECs with CHIR-99021 (an inhibitor of GSK-3β) had an accelerative effect on the CIEC proliferation. Meanwhile, we examined apoptosis-related protein expression in vitro. As shown in Figure 9G–I, treatment with LY294002 (PI3K inhibitor), GSK-690693 (AKT inhibitor), CHIR690626 (GSK-3β inhibitor), 4-CMTB (GPR43 agonist) and PTX (Gi inhibitor), resulted in the upregulation of the pro-apoptosis protein Bax, Caspase-3 expression and the Bax/Bcl-2 ratio but also downregulated the expression of the anti-apoptosis protein Bcl-2 compared with the butyrate group. However, AR420626 (GPR41 agonist) and Ym254890 (Gq inhibitor) showed no significant difference in the protein expression of Bax, Caspase-3, Bcl-2, or the Bax/Bcl-2 ratio. Hence, butyrate exerted its pro-proliferation and anti-apoptosis effects through the GPR43/Gi/PI3K/AKT/GSK-3β/β-catenin/cyclin D1 signaling pathway.

## 4. Discussion

In recent years, a series of studies reported that microbes strongly react to light and that the fungi metabolism process is sensitive to light response [23,24]. Light pollution, which is caused by artificial lighting at night, leads to changes in the composition of gut microbes [25]. Our research suggested that BL light could effectively improve the richness and diversity of the jejunal microbiome, which is beneficial for the intestinal health of chicks. Several reports have shown that reduced gut microbial diversity is associated with obesity-related diabetes and inflammatory bowel disease [26,27,28]. Additionally, we found that more than 98% of the bacterial genes were derived from four phyla, including *Firmicutes, Actinobacteria, Proteobacteria,* and *Bacteroides*, in the jejunums of chicks, similar to previous observations in chicks [29]. However, there were still minor differences between WL, RL, GL and BL. In WL, *Firmicutes* was predominant while *Bacteroidetes, Proteobacteria* and *Actinobacteria* accounted for a smaller percentage in the chick jejunum gut catalogs. However, in GL, RL and BL, the relative abundance of *Firmicutes* was decreased and the relative abundance pf *Bacteroidetes, Proteobacteria* and *Actinobacteria* were increased. These changes in the phylum may lead to functional differences. A previous study showed that the upregulated abundance of *Bacteroides* could enhance the potential of microbial glycolysis in chicks [30] and highly enriched *Proteobacteria* may be related to the promotion of microbial glycolysis to obtain energy [31]. Thus, a previous study suggested that different monochromatic light can affect the composition and diversity of intestinal microorganisms. However, the effect of different light wavelengths on the intestinal microbiome composition is also related to species and the location of the digestive tract. In Peking ducks, when imposing low-light intensity blue or green monochromatic light, the amount of *Firmicutes* increased but the amount of *Bacteroidetes* decreased in the cecum, compared to white light [32].

At the genus level, compared with WL, RL significantly decreased the relative abundance of *Paracoccus*, which was closely related to the digestion and absorption of nitrogen and carbon [33]. Moreover, green light decreased the relative abundances of *Blautia*, which led to increased intestinal permeability [34], as well as that of *Staphylococcus*, which can damage the intestinal epithelial barrier [35]. BL significantly increased the relative abundances of *Alistipes, Anaerofilum, Bacteroides, Faecalibacterium* and *Ruminiclostridium_9*. *Bacteroides*, which is a beneficial bacteria correlated with short-chain fatty acids production, is involved in many important metabolic activities, including the induction of key glycolytic enzymes in intestinal epithelial cells and the fermentation of carbohydrates [36,37]. Moreover, *Alistipes* [38], *Faecalibacterium* [39], *Anaerofilum* [40] and *Ruminiclostridium_9* [41] are all involved in producing short-chain fatty acids, which is possibly helpful to microalgal growth and intestinal health. These results demonstrated that microbes responding to different wavelengths of light are largely dissimilar; we speculated that the metabolites of the jejunal content may change under different light regimes. To further verify our hypothesis, we used gas chromatography to detect the effect of different monochromatic lights on short-chain fatty acid concentrations in the jejunum. The results showed that BL significantly improved the production of butyrate, which may have been caused by the greatly increased amounts of bacteria such as *Faecalibacterium* and *Ruminiclostridium_9*. These specific taxa enriched in different monochromatic light treatments indicated an early change in energy metabolism profile and the enrichment of beneficial gut microbiota in blue light. Consistent with our suggestions, Alsanius et al. reported that blue light influenced the substrate utilization patterns for the nutrients of non-phototrophic bacteria [42].

A previous study proposed that the “core” microbial community in the gut is not determined by the actual species of bacteria, but instead by the collective functional characteristics contained in the community [43]. It was interesting to note that the effects of BL on the functional capabilities of the microbial communities were similar to the observation on *amino acid and glucose transporter* mRNA levels. The KEGG difference analysis showed that there was a higher abundance of genes involved in metabolism (such as the biosynthesis of amino acids, or carbon metabolism) enriched in BL compared to WL. Our previous laboratory study demonstrated that blue light improved body weight and growth performance in the later stage [44]. Meanwhile, Church et al. suggested that amino acid biosynthesis is crucial for increasing muscle and body protein synthesis [21], as well as carbon metabolism, which is necessary to maintain body health and redox defense [45]. Therefore, we supposed that the difference in the predicted microbial function may lead to altered nutrient availability and help to explain why the use of blue light resulted in improved chick growth performance.

BL significantly improved the antioxidant capacity of the jejunum and reduced ROS production. Abdo et al. also reported that 480 nm blue light could reduce fear and heat stress levels in chicks after hatching [46]. A previous study suggested that decreases in intestinal microbial diversity can be caused by the oxidative stress that occurs during inflammation. In addition, the aggravation of oxidative stress can promote the growth and amplification of some specific bacterial groups [47]. Our Spearman’s correlation analysis also showed that the ACE index and the Shannon index were positively correlated with antioxidant enzyme activity and negatively correlated with pro-inflammatory levels. Therefore, our findings implied that BL alleviated oxidative stress and suppressed the production of pro-inflammatory cytokines, which alter the composition of microorganisms.

Interestingly, we also found that BL significantly promoted intestinal development and reinforced epithelial barrier functions by increasing the V/C ratio and the number of PCNA, MUC2 and goblet cells, as well as enhancing tight jejunal junctions and showing low intestinal permeability. Studies have shown that accelerated intestinal development can improve the digestive and absorption capacity of intestinal nutrients [48]. A positive correlation between tight jejunal junctions and the abundance of *Romboutsia* was observed, suggesting that the rapid development of the intestinal tract provides a good colonization environment for microorganisms. There was a positive correlation between the *SI* mRNA level and the abundance of *Romboutsia*, indicating that digestion capacity was also affected by the intestinal microbiota. Overall, these findings suggested that BL could significantly improve jejunal digestion and absorption ability and that the intestinal microbiota has a variety of interactions, both in nutrient exchange and in the physiology of the digestive system.

Our study found that 480 nm blue light could effectively increase the diversity and richness of jejunal microbes and improve *Faecalibacterium* and *Ruminiclostridium_9*. Consistent with these results, the gas chromatography assays showed that blue light increased jejunal butyrate concentrations but had no effect on the acetate and propionate concentration in the jejunum. Because *Faecalibacterium* [49] and *Ruminiclostridium_9* [50] are both involved in producing butyrate, we supposed that BL significantly improved the production of butyrate, which may have been caused by the greatly increased amounts of bacteria, such as *Faecalibacterium* and *Ruminiclostridium_9.* In addition, we observed that blue light improved intestinal mucosal barrier integrity, reduced the oxidative level and promoted small intestine development. In recent years, emerging data demonstrated that the gut microbiota plays an important role in protecting the integrity of the epithelial barrier, forming the mucosal immune system, and maintaining intestinal homeostasis through its metabolites, particularly butyrate. Butyrate, as an important energy source for intestinal epithelial cells, can promote gut morphology development [51], enhance barrier function [52] and decrease oxidative stress in chicks [53]. Therefore, we speculated that microorganisms and their metabolites may play an important role in mediating blue-light-induced intestinal development and mucosal barrier integrity. To confirm our hypothesis, we added exogenous butyrate to small intestinal epithelial cells in vitro. The in vitro experiments showed that butyrate-induced CIECs proliferation was mediated by GPR43, but not GPR41. Yang et al. also found that butyrate promotes Th1 cell differentiation via GPR43 pathways [54]. Furthermore, we found that butyrate could promote CIEC proliferation, which was scarcely influenced by the Gi inhibitor PTX and not blocked by the Gq inhibitor Ym254890. This result is consistent with a previous report showing that GPR43 is a Gi/o and Gq-coupled GPCR, but that its functions are mainly mediated by Gi/o [55]. In our study, we found that the PI3K/AKT, GSK-3β and β-catenin cascades may be involved in blue-light-induced, GPR43-mediated CIEC proliferation. A previous report also indicated that butyrate can activate GSK-3beta phosphates, causing cell proliferation and differentiation [56]. In addition, goblet cell differentiation and barrier integrity could also be regulated by microorganisms and their metabolites. A previous study found bacterial component LPS can induce goblet cell to secret MUC2 [57]. Furthermore, the bacterial metabolite butyrate can enhance the intestinal barrier and facilitate tight protein expression by activating AMP-related protein [58] and induce intestinal stem cell differentiation through a Foxo3-dependent mechanism [59]. Overall, these results indicated that blue light may change the composition of jejunal microbes, leading to changes in the concentration of butyrate, ultimately affecting intestinal development and mucosal barrier integrity. However, in addition to butyrate, other types of intestinal microbiota metabolite also play important roles in mediating the signaling from intestinal microorganisms to epithelial cells, such as secondary bile acids [60] and tryptophan [61]. Therefore, fecal microbiota transplantation or butyrate supplementation tests should be carried out in the future to verify the core role of intestinal microbes in butyrate-mediated, blue-light-induced small intestinal development and mucosal barrier function enhancement.

## 5. Conclusions

In summary, the present study provided evidence that blue light used in poultry production can reduce oxidative stress levels and inflammatory response. This protection against oxidative stress leads to increased diversity and richness in the jejunum, and increases the relative abundance of *Faecalibacterium*, *Ruminiclostridium_9* and its metabolite, butyrate. Butyrate may act as one of the signals to mediate blue-light-induced small intestine development and mucosal barrier integrity enhancement. In addition, we revealed that pretreatment with butyrate effectively promoted cell proliferation and inhibited cell apoptosis through the GPR43/Gi/PI3K/AKT/GSK-3β/β-catenin pathway in chick small intestinal epithelial cells.

## Figures and Tables

**Table 1:** Ingredients and composition of basal diet (%, dry matter basis).

Ingredients	Content
Dry matter	86.05
Crude protein	19.09
Crude ash	8.04
Sodium chloride	0.67
Calcium	0.85
Phosphorus	0.42
Methionine + Cystine	0.71

**Table 2:** Sequences of primers used for RT-PCR.

Gene	Product Size	Primer Sequences (5′–3′)	Accession No.
*PepT1*	180	F: TTTCCTTTACATCCCTCTCCCG R: ATCACAGCATCTACAACTGGGACC	AY029615
*SI*	132	F: TGGATTGTCATCACCCGTTC R: CCAAAGAGACTGAACTCCATCATACC	XM_422811
*SGLT1*	124	F: TGGCGGGCTTCTACCGCAGCGAG R: CCCGGTAGGTCACCAGTCCCCAG	XM_415247
*GLUT2*	107	F: GCCTTGAGGAAACATCTGCT R: GGACTGGATGGACGTTATGG	Z22932
*CAT-1*	127	F: TCTGCTCATCTGCTTTGTGG R: GGCTCCATCCCAACCTACAT	XM_417116
*CAT-2*	106	F: GCTAACTTGGAGCCCTGGAG R: CCACTTTCTGCTGGTTCTGC	XM_420685
*GPR43*	149	F: AACGCCAACCTCAACAAGTC R: TGGGAGAAGTCATCGTAGCA	NM_001318430.1
*GPR41*	103	F: GAAGGTGGTTTGGGAGTGAA R: CAGAGGATTTGAGGCTGGAG	XM_040693461.1
*β-actin*	169	F: TCCACCGCAAATGCTTCTAAAC R: CTGCTGACACCTTCACCATTCC	NM_205518

F = forward primer; R = reverse primer.

## Data Availability

The sequencing data we generated were deposited in the NCBI Sequence Read Archive (SRA) under accession numbers from SAMN19812657 to SAMN19812672 in PRJNA739905.
